# Calibration of Novel Protein Biomarkers for Veterinary Clinical Pathology: A Call for International Action

**DOI:** 10.3389/fvets.2019.00210

**Published:** 2019-07-02

**Authors:** Peter David Eckersall

**Affiliations:** ^1^Institute of Biodiversity, Animal Health and Comparative Medicine, College of Medical, Veterinary and Life Sciences, University of Glasgow, Glasgow, United Kingdom; ^2^European Research Area (ERA) Chair Laboratory, VetMedZg, Faculty of Veterinary Medicine, University of Zagreb, Zagreb, Croatia

**Keywords:** biomarker, acute phase, protein, calibration, standardization and certification systems

## Abstract

Research into the identification and use of protein biomarkers for use in veterinary clinical pathology has produced numerous potential analytes that could become common tests in the future. One problem that has to be overcome in the general acceptance of a novel biomarker is that differing standards for calibration may be developed by individual laboratories or the diagnostic companies that will provide kits for widespread use. This has been apparent in the development of acute phase protein biomarkers such as canine C-reactive protein. In order to overcome this problem an international initiative is required to ensure that assays developed in separate laboratories would have a consistent calibration protocol so that results produced are equivalent. International reference preparations for serum protein analysis for each relevant species should be established for use as primary standard in the calibration of biomarkers for veterinary diagnosis.

## Introduction

Biomarkers are defined by the Biomarker Working Group of the National Institutes of Health Director's Initiative on Biomarkers and Surrogate Endpoints as “A characteristic that is objectively measured and evaluated as an indicator of normal biological processes, pathogenic processes, or pharmacologic responses to a therapeutic intervention” (1). This covers many if not all analytes in diagnostic laboratories, however in veterinary clinical pathology, the major recent biomarker developments which have been valuable for improving the diagnosis of disease in domestic animals have been in the identification, validation and assay development for protein biomarkers especially in serum, or plasma.

Over the last few decades, there have been investigations into a number of protein biomarkers the concentration of which have the potential for use in diagnostic laboratories and potentially for point-of-care testing in veterinary practices. Thus, proteins such as, C-reactive protein, serum amyloid A, α_1_ acid glycoprotein, adipokines, cytokines, telomerase, paroxonase, pancreatic lipase, GLUT1, cardiac troponin, brain natriuretic peptide, VCAM-1 and LCAM1 are already in use or their potential has been identified for monitoring a wide variety of conditions including innate immunity, neoplastic disease and cardiac medicine in both companion and production animals. Tremendous strides have been made in the discovery and application of veterinary biomarkers and these have been reviewed elsewhere (2–4).

However, there is a major hurdle to overcome in the implementation of novel biomarkers for veterinary diagnosis, especially for methods that could be implemented around the world. There is no international agreement for harmonization of assay calibration for veterinary biomarkers. This means that there is no accepted means to calibrate a specific assay for a novel biomarker. Therefore, a potential problem can arise in provision of consistent results and alignment of clinical interpretation, especially if two or more laboratories use differing calibration standards. If a globally agreed protocol can be developed to overcome this shortcoming in veterinary clinical pathology, then all laboratories and of equal importance all diagnostic kit manufacturers would have access to the same accepted standard material for calibration so that results would then be comparable between methods.

Before outlining a solution to this problem it is instructive to illustrate the means whereby biomarkers have been developed in the recent past. For example, following the development of assays for canine C-reactive protein (cCRP) over the last 40 years illustrates how problems can arise. Current technological approaches can accelerate the discovery of further biomarkers and integral to further advances would be a regulatory system for international harmonization of the standards used in assay calibration. It is also instructive to illustrate how standards for calibration have been developed for clinical biochemistry in human medicine.

## Biomarker Development

The incentive to establish a novel biomarker has varied origins. It may be by a deliberate investigation to identify a biomarker for a specific disease, it may be a serendipitous discovery of a protein that can be the target for a new biomarker test, or it may be by comparison to established biomarkers in human medicine. It was the latter that formed the basis for the development of cCRP as a marker of inflammation, infection and trauma. Identified as a serum protein in the 1920's the use of this biomarker in human medicine was established in the 1970s and today is one of the most frequently assayed analytes in medical hospitals and general practice. Although CRP had been identified in canine serum during an acute phase response (5) its use as a biomarker in veterinary medicine was not documented until assay methods for use in diagnostic laboratories were established independently in Japan and UK (6, 7). A variety of immunoassay formats including radial immunodiffusion, ELISA and immunoturbidimetry were established in veterinary research laboratories. Unfortunately, at this early stage a common calibration standard was not developed in the active research laboratories. However, the clinical utility of the use of cCRP as a marker for disease in dogs developed because in-house assays used in research studies were able to identify the best applications (8) of this acute phase protein test. The tests for cCRP, as it is elevated by many causes, were found to be especially valuable in identification of infectious and inflammatory disease, with quantification being important to reveal the extent of pathology and to monitor responses to treatment (9).

Encouraged by the initial findings and by a growing network of specialist centers the clinical uses of the test became established with major application by laboratories such as in Murcia (10), Copenhagen (11), in Europe and Hokkaido in Japan (6). Outside the main research laboratories further use of cCRP was enabled by the finding that some but not all assay kits for human CRP could be used to measure the canine protein (12). At an earlier stage in Japan than in Europe, commercially produced kits for determining cCRP concentrations in serum or plasma were developed which led to a widespread uptake of cCRP analysis in veterinary practices in Japan. Now a number of cCRP assay kits both for laboratory analysis and for point-of-care use have been developed by differing manufacturers in Europe and the USA.

The more ready access now available by veterinarians in practice to cCRP assays has led to a virtuous cycle in that the greater availability is leading to more research and more defined applications. Indeed the full extent of the uses of cCRP as a marker of innate immunity in dogs is probably not fully exploited and will benefit from multi-centric determination of benefits and limitations. This is highlighting that it is essential for comparison between research results from different laboratories that a common basis is used for calibration of cCRP assays. Then reference ranges, clinical decision point and clinical findings will be similar whichever diagnostic kit is utilized in a clinical biochemistry laboratory or in practice point-of-care instrument.

The development, validation and use of cCRP has therefore been a 30 year process. Current develops in the discovery of novel biomarkers has been accelerating, but for implementation of other new tests will face similar barriers as have been encountered for cCRP. Thus, it is opportune to develop a process that would be applicable to all new assays for veterinary biomarkers, not only for biomarkers in serum but also with the exciting prospects for monitoring disease with non-invasive samples for instance by use of biofluids such as saliva (13–15), or milk (16, 17) as the matrix for examination.

A major driver to the discovery of new veterinary biomarkers is the application of recent advances in proteomics using liquid chromatography and mass spectrometry (18) so making it more imperative that a process is developed to deal with calibration standardization. In relevant example in dogs, such advanced technology has identified biomarkers associated with canine babesiosis (19) while in another study biomarkers for treatment monitoring of canine leishmaniosis (20) have been discovered. Feline biomarkers have also been investigated by proteomic study in research on familial hypertrophic cardiomyopathy (21). In farm animal medicine the use of proteomics has generated substantial interest in biomarker development, especially for disease such as bovine mastitis where an on-farm biomarker test could be particularly valuable (22). For global utilization of such advances, harmonization of assay calibration will be important, if not essential.

## Calibration for Reference Standards for Protein Biomarkers

For the majority of assays currently employed in veterinary clinical biochemistry, their calibration is well-established. For small molecules such as ions, metabolites such as urea and glucose and steroid hormones, the molecules are identical across species. For enzymes such as alkaline phosphatase their activity is measured by the same enzyme reactions in all species so that calibration does not present a problem. Similarly albumin and total protein are measured by chemical reactions common in all species and their calibration is well-established.

Specific protein biomarkers present a different problem as the proteins often vary across different species due to differences in their genome and protein conformation. Furthermore, differences in the matrix of the sample between species may, interacting with species specific protein, cause differences in the calibration of assays. Therefore, when measured by immunoassay, the method of choice for most protein biomarkers, antibody reactions will differ between species and there is also variation between the reactions of antibody from differing sources. Furthermore, a gold standard calibrator of a pure sample of the protein biomarker may only be available by tedious purification procedures which commonly do not yield preparations of 100% purity. Recombinant protein or synthesized peptides can be used for production of calibration standards either polyclonal or monoclonal antibody and calibration standards can be based such synthesized protein but they may not react as native protein. For instance glycosylation may be absent in synthesized protein or the native conformation may not be achieved reducing antibody interaction. These features mean that assays based on purified protein or recombinant protein may give different calibrations when used in immunoassay standard curves. A freely available calibration standard for use in assay development would overcome these hurdles to biomarker development.

The problem that can arise from the lack of an internationally recognized primary calibration standard preparation can be illustrated by the study conducted by Munoz-Prieto et al. (12) in which canine serum with differing concentrations of cCRP were used as quality controls (QC) to determine the precision of four different commercially available assays for this biomarker. In assessment of the interassay coefficient of variance (CV) the high level QC sample gave acceptable CV values for all four assays of < 12.86% but the mean ± SD values varied by 50% ranging from 97 ± 12 mg/L to 150.8 ± 14.3 mg/L^1^. For the medium level QC sample CVs were all <11% but the mean values varied by 240% ranging from 28.7 ± 3.1 to 70.4 ± 12.5 mg/L. For the low QC all the CVs were <15% but the mean values varied by 383% ranging from 3.18 ± 0.19 mg/L to 12.2 ± 1.28 mg/L on the same QC sample. Canine CRP reference ranges based on samples from healthy dogs in the same region (~10 mg/L) as the lowest of these QC samples (7, 23). A difference of approaching 400% between assays is not ideal and could lead to false negative or false positive results if a range from one assay were to be used for result interpretation from another cCRP assay method.

There have been few if any similar investigations comparing assays for other novel protein biomarkers, however there has been recent studies of results from assays of bovine haptoglobin in investigation of bovine respiratory disease that illustrate the problem caused by lack of an international calibration standard. A meta analysis of papers in the literature reporting on the use of bovine haptoglobin as a biomarker of this condition (24) identified 314 studies although only 23 met inclusion criteria for the meta analysis. Of these 52% of the investigations used an ELISA method from one manufacturer with a consistent calibration standard which gave reference range in healthy cattle of 0–0.15 mg/ml^1^ with a median haptoglobin concentration of 2.6 mg/ml in cattle with bovine respiratory disease (25). In contrast another recent investigation (26) using an ELISA from a different manufacturer described the serum concentration of haptoglobin in healthy cows as being <10 ng/ml^1^ with serum concentrations 115–163 ng/ml in animals with bovine respiratory disease thus showing a 10^4^ difference in the reported values in the disease cases between results obtained by different commercially available assays. It is important that such variations are eliminated especially future development of novel protein biomarkers as they transfer from research laboratories to use in clinical assessment.

Similar problems have been encountered and overcome by scientists in human clinical biochemistry. Solutions have been applied by international agreement to the benefit of the diagnostic industry so that whichever method and manufacturer's product is used, specific protein assay kits are calibrated against a common standard which is openly available to any producer with the result that all assays for a particular serum biomarker should give a similar result.

Collaborative initiatives under the auspices of the International Federation of Clinical Chemistry (IFCC) set a precedent that has been in place for over three decades. In 1994 the IFCC established an international Reference Preparation for Proteins in Human Serum (RPPHS) (27, 28) which provided a pool of human serum with a known concentration of the major biomarkers in the serum determined as the mean value of all laboratories in the collaboration. This has been updated with the most recent version being ERM-DA470k (29, 30). In veterinary science, there was an initiative to generate similar reference preparations for calibration of acute phase protein in bovine and porcine serum (31) and these are still available from the University of Copenhagen and University of Zaragosa, respectively. Although these reference preparations have been available knowledge of their existence was not widely disseminated and in future it will be important that all potential users should be aware of the availability of such preparations.

However, not all protein assays for use in veterinary clinical pathology present a problem in calibration. For instance, assays for canine troponin I (cTnI) the cardiac biomarker, are often based on antibodies produced against the human cTNI protein but the high degree of conservation of cTnI in structure across species (32) enables the use of these assays in canine cardiac cases with calibration against the human protein. Such assay development where the human calibrant is well-established and validated or where there is agreement among diagnostic kit manufacturers on the nature of the calibrator, would not need further attention.

## A Proposal for International Harmonization of Veterinary Biomarker Calibration

Procedures for developing these reference preparations for assays, where they are needed, are described in Table 1 and involves the following protocol for calibrators for assays for a specific species. A pool of serum of the species should be prepared containing the analyte being measured at a concentration aligning with the upper region of standard curves in use. The pool would be made by or transferred to the coordinating laboratory. Aliquots would be distributed to laboratories where the relevant protein biomarker assays are in use and the laboratories will analyse serum for the biomarkers of choice. Results will be returned to a central data analyst at the coordinating laboratory with details of the assay method and kit manufacturer. An all laboratory mean for each protein in the serum will be calculated and all partners in association with the scheme will reach agreement on the mean value of the protein biomarker which will then be used as primary standard for calibration of assays and diagnostic kits. A stock of the standard will be stored securely at −80°C in at least 3 locations and in aliquots that can be distributed on request to any laboratory and diagnostic manufacturer for use as a laboratory primary standard against which working standards can be calibrated.

**Table 1:** A protocol for preparation and standardization of biomarker calibrators.

1. Prepare a pool of serum suitable for the programme.
2. Dispatch aliquots to laboratories where the protein biomarker assays are in use.
3. Each laboratories will analyse serum for the biomarkers of choice.
4. Results will be returned to a central data analyst at the coordinating laboratory with details of the assay method and kit manufacturer.
5. The all laboratory mean for each protein biomarker in the serum will be determined.
6. After agreement on the mean value of each protein biomarker the pooled serum will be used as a primary calibrator of all assays.
7. The pooled serum will be stored securely at 3 locations at −80°C in aliquots and made available to any laboratory and diagnostic manufacturer for use as a laboratory primary standard against which working standards can be calibrated.

The current climate for developing veterinary biomarkers with increased recognition of their potential value in diagnosis, and the increasing involvement of commercial organizations developing diagnostic testing for the global community, make this an opportune time for there to be an international initiative for addressing the problem of a lack of veterinary reference preparations for animal species. The requirement for species specific reference preparations but would in the first instance focus on canine and feline serum proteins to supplement the previous development of bovine and porcine serum preparations.

The International Society of Animal Clinical Pathology (ISACP), as a society with international reach has agreed to sponsor this process by initiating a working group to define a clear plan of how this can be facilitated. The working group would be composed of veterinary academic and diagnostic industry representatives including representatives from national and supra-national organizations in the field, such as the American College of Veterinary Clinical Pathology, the Japanese College of Veterinary Clinical Pathology, the European College of Veterinary Clinical Pathology and other related organizations. The Working Party would be charged with the responsibility to prepare a detailed plan for establishing International Veterinary Biomarker Reference Preparations. An outline of the Working Party proposal is given in Table 2 and consists of the following. The Working Party should have members from the major national/regional societies where these exist covering veterinary clinical pathology in North America, Japan and Europe and also from national groups where such societies are not present. There should be members from diagnostic companies involved in the preparation of veterinary biomarker diagnostic kits. There should also members who have the technical skill and experience to oversee the preparation, distribution and analysis of results from calibrator standardization. The working party should develop a standard operating procedure to be used in preparing an international reference preparation for each of the major veterinary species. Laboratories capable of participating in the development of the calibrators will be identified and a coordinator of the process confirmed. Funding will be required to undertake this programme and could be sought from industry, who will benefit directly from having consistent and recognized biomarker calibration, academia via grant application to national and international funding agencies and societies devoted to improving animal health and welfare.

**Table 2:** A proposal for an international working party for standarization of calibration of veterinary biomarkers.

**Formation of the working party**
1.Members delegated from ISACP, JSVCP, ESVCP, ASVCP, and other national associations in order to relate discussion to their members.
2.Members from industry to liaise with all veterinary biomarker companies.
3.Experts appointed in protein biomarker analysis, including scientific (protein biochemistry, molecular biology), in veterinary clinical pathology and in statistical analysis.
**Initial activity of the working party**
4.Identify biomarkers where standards are required.
5.Define standard operating procedures for preparation analysis and distribution of international reference material for veterinary biomarker assays.
6.Identify participating laboratories and a coordinating center.
7.Seek funding from industry and academia—including experts in metrology, Institute for Reference Material and Measurements for production of European Reference Material, Institute for Biological Standards.

## Conclusion

The development and widespread use of veterinary protein biomarkers is increasing but for regular use by diagnostic laboratories across the world it is important that methods use common reference preparations for calibration of standards and generation of standard curves for quantification of the biomarkers in clinical samples. In order to enable the development of species specific reference preparations an international harmonization programme is proposed that will allow assays to be developed to a global calibration level so that there should be comparable results obtained from investigation of a similar sample whichever assay method or diagnostic kit is used. The nature and extent of such a programme is open for debate among clinical pathologists in research, clinical laboratories and industry with the ultimate aim of improving and harmonizing biomarker assays for veterinary diagnosis.

## Author Contributions

The author confirms being the sole contributor of this work and has approved it for publication.

### Conflict of Interest Statement

The author declares that he has had research links and advisory roles with commercial companies producing kits for veterinary diagnostic laboratories.

## Footnotes

^1^Units are used as in the papers quoted.

## References

[1] Atkinson AJ Colburn WA DeGruttola VG DeMets DL Downing GJ Hoth DF Biomarkers and surrogate endpoints: Preferred definitions and conceptual framework. Clin Pharmacol Ther. (2001) 69:89–95. 10.1067/mcp.2001.113989 11240971

[2] Moore RE Kirwan J Doherty MK Whitfield PD. Biomarker discovery in animal health and disease: the application of post-genomic technologies. Biomark Insights. (2007) 2:185–196. 10.1177/117727190700200040 19662203 PMC2717813

[3] Mobasheri A Cassidy JP. Biomarkers in veterinary medicine: Towards targeted, individualised therapies for companion animals. Vet J. (2010) 185:1–3. 10.1016/j.tvjl.2010.04.003 20541693

[4] Myers MJ Smith ER Turfle PG. Biomarkers in Veterinary Medicine. Annu Rev Anim Biosci. (2017) 5:65–87. 10.1146/annurev-animal-021815-111431 27860493

[5] Caspi D Snel FWJJ Batt RM Bennett D Rutteman GR Hartman EG. C-reactive protein in dogs. Am J Vet Res. (1987) 48:919–21. 3605808

[6] Yamamoto S Shida T Okimura T Otabe K Honda M Ashida Y. Determination of C-reactive protein in serum and plasma from healthy dogs and dogs with pneumonia by ELISA and slide reversed passive latex agglutination test. Vet Q. (1994) 16:74–7. 10.1080/01652176.1994.9694422 7985359

[7] Eckersall PD Conner JG Parton H. An enzyme-linked immunosorbent assay for canine C-reactive protein. Vet Rec. (1989) 124:490–1. 2750033 10.1136/vr.124.18.490

[8] Ceron JJ Eckersall PD Martínez-Subiela S. Acute phase proteins in dogs and cats: current knowledge and future perspectives. Vet Clin Pathol. (2005) 34:85–99. 10.1111/j.1939-165X.2005.tb00019.x 15902658

[9] Eckersall PD Bell R. Acute phase proteins: biomarkers of infection and inflammation in veterinary medicine. Vet J. (2010) 185: 23–7. 10.1016/j.tvjl.2010.04.009 20621712

[10] Ceciliani F Ceron JJ Eckersall PD Sauerwein H. Acute phase proteins in ruminants. J Proteomics. (2012) 75:4207–31. 10.1016/j.jprot.2012.04.004 22521269

[11] Nielsen L Toft N Eckersall PD Mellor DJ Morris JS. Serum C-reactive protein concentration as an indicator of remission status in dogs with multicentric lymphoma. J Vet Intern Med. (2007) 21:1231–6. 10.1892/07-058.1 18196731

[12] Alberto MP Asta T Damián E Silvia MS José JC Use of heterologous immunoassays for quantification of serum proteins: the case of canine C-reactive protein. PLoS ONE. (2017) 12:1–14. 10.1371/journal.pone.0172188 PMC5319752 28222144

[13] Parra MD Tecles F Martinez-Subiela S Ceron JJ. C-reactive protein measurement in canine saliva. J Vet Diagn Invest. (2005) 17:139–144. 10.1177/104063870501700207 15825494

[14] Gutiérrez AM Nöbauer K Soler L Razzazi-Fazeli E Gemeiner M Cerón JJ. Detection of potential markers for systemic disease in saliva of pigs by proteomics: a pilot study. Vet Immunol Immunopathol. (2013) 151:73–82. 10.1016/j.vetimm.2012.10.007 23177629

[15] Gutiérrez A Cerón JJ Razzazi-Fazeli E Schlosser S Tecles F. Influence of different sample preparation strategies on the proteomic identification of stress biomarkers in porcine saliva. BMC Vet Res. (2017) 13:1–11. 10.1186/s12917-017-1296-9 29202764 PMC5716369

[16] Thomas FC Waterston M Hastie P Parkin T Haining H Eckersall PD. The major acute phase proteins of bovine milk in a commercial dairy herd. BMC Vet Res. (2015) 11:207. 10.1186/s12917-015-0533-3 26276568 PMC4536752

[17] Pyörälä S Hovinen M Simojoki H Fitzpatrick J Eckersall PD Orro T. Acute phase proteins in milk in naturally acquired bovine mastitis caused by different pathogens. Vet Rec. (2011) 168:535. 10.1136/vr.d1120 21558129

[18] Bilic P Kules J Galan A de Pontes LG Guillemin N Horvatic A Proteomics in veterinary medicine and animal science: Neglected Scientific opportunities with Immediate Impact. Proteomics. (2018) 1800047:1–7. 10.1002/pmic.201800047 29952133

[19] Kuleš J Mrljak V Rafaj RB Selanec J Burchmore R Eckersall PD. Identification of serum biomarkers in dogs naturally infected with Babesia canis canis using a proteomic approach. BMC Vet Res. (2014) 10:111. 10.1186/1746-6148-10-111 24885808 PMC4045879

[20] Martinez-Subiela S Horvatic A Escribano D Pardo-Marin L Kocaturk M Mrljak V. Identification of novel biomarkers for treatment monitoring in canine leishmaniosis by high-resolution quantitative proteomic analysis. Vet Immunol Immunopathol. (2017) 191:60–7. 10.1016/J.VETIMM.2017.08.004 28895868

[21] Meurs KM Sanchez X David RM Bowles NE Towbin JA Reiser PJ. A cardiac myosin binding protein C mutation in the Maine Coon cat with familial hypertrophic cardiomyopathy. Hum Mol Genet. (2005) 14:3587–93. 10.1093/hmg/ddi386 16236761

[22] Mudaliar M Tassi R Thomas FC McNeilly TN Weidt SK McLaughlin M. Mastitomics, the integratedomics of bovine milk in an experimental model of: *Streptococcus uberis* mastitis: 2. Label-free relative quantitative proteomics. Mol Biosyst. (2016) 12:2748–61. 10.1039/c6mb00290k 27412694 PMC5048399

[23] Ohno K Yokoyama Y Nakashima K Setoguchi A Fujino Y Tsujimoto H. C-reactive protein concentration in canine idiopathic polyarthritis. J Vet Med Sci. (2006) 68:1275–79. 10.1292/jvms.68.1275 17213695

[24] Abdallah A Hewson J Francoz D Selim H Buczinski S. Systematic review of the diagnostic accuracy of haptoglobin, serum amyloid A, and fibrinogen versus clinical reference standards for the diagnosis of bovine respiratory disease. J Vet Intern Med. (2016) 30:1356–68. 10.1111/jvim.13975 27255433 PMC5089617

[25] Wolfger B Schwartzkopf-Genswein KS Barkema HW Pajor EA Levy M Orsel K. Feeding behavior as an early predictor of bovine respiratory disease in North American feedlot systems. J Anim Sci. (2015) 93:377–85. 10.2527/jas.2013-8030 25568380

[26] Joshi V Gupta VK Bhanuprakash AG Mandal RSK Dimri U Ajith Y. Haptoglobin and serum amyloid A as putative biomarker candidates of naturally occurring bovine respiratory disease in dairy calves. Microb Pathog. (2018) 116:33–7. 10.1016/j.micpath.2018.01.001 29330058

[27] Whicher JT Ritchie RF Johnson AM Baudner S Bienvenu J Blirup-Jensen S A Clinical Chemistry. New international reference preparation for proteins in human serum (RPPHS). Clin Chem. (1994)40:934–8. 8087989

[28] Whicher JT. BCR/IFCC reference material for plasma proteins. Clin Biochem. (1998) 31:459–65. 9740967 10.1016/s0009-9120(98)00035-6

[29] Zegers I Schreiber W Linstead S Lammers M McCusker M Muñoz A. Development and preparation of a new serum protein reference material: feasibility studies and processing. Clin Chem Lab Med. (2010) 48:805–13. 10.1515/CCLM.2010.166 20374041

[30] Zegers I Keller T Schreiber W Sheldon J Albertini R Blirup-Jensen S. Characterization of the new serum protein reference material ERM-DA470k/IFCC: value assignment by immunoassay. Clin Chem. (2010) 56:1880–88. 10.1373/clinchem.2010.148809 20923953

[31] Skinner JG. International standardization of acute phase proteins. Vet Clin Pathol. (2001) 30:2–7. 10.1111/j.1939-165X.2001.tb00248.x 12024323

[32] Porter A Rozanski E Price LL Shaw S. Evaluation of cardiac troponin I in dogs presenting to the emergency room using a point-of-care assay. Can Vet J. (2016) 57:641–5. 27247465 PMC4866670

